# Super‐Resolution Imaging With Fluorotellurite Glass Microspheres

**DOI:** 10.1002/nap2.70041

**Published:** 2026-02-27

**Authors:** Haonan Zhuo, Shengchuang Bai, Zhouyi Yu, Zhenmin Wang, Zejie Zheng, Yu Zhuang, Yina Jiang, Tianyao Zhang, Hao Li, Lixiang An, Duanduan Wu, Xunsi Wang, Hui Yang, Guoqiang Gu

**Affiliations:** ^1^ Shenzhen Institutes of Advanced Technology Chinese Academy of Sciences Shenzhen China; ^2^ Laboratory of Infrared Material and Devices Advanced Technology Research Institute Ningbo University Ningbo China; ^3^ College of Physics and Optoelectronic Engineering Shenzhen University Shenzhen China; ^4^ National Innovation Center for Advanced Medical Devices Shenzhen China; ^5^ Stay Key Laboratory of Biomedical Imaging Science and System, Key Laboratory of Biomedical Imaging Science and System Chinese Academy of Sciences Shenzhen China; ^6^ University of Chinese Academy of Sciences Beijing China

**Keywords:** fluorotellurite glass, microsphere, super‐resolution imaging, ultramicroscopic objective

## Abstract

Microsphere‐lens‐assisted optical nanoscopy has emerged as a powerful approach for surpassing the diffraction limit of conventional optical microscopy. Here, we present a comprehensive investigation of high‐refractive‐index fluorotellurite (TeO_2_‐BaF_2_‐Y_2_O_3_, TBY) glass microspheres fabricated by a high‐temperature floating‐zone melting technique. The microspheres exhibit excellent sphericity, ultra‐smooth surfaces, diameters from 10 to 200 μm, a refractive index of ∼1.9, and up to 85% visible transmittance. Ray‐tracing and full‐wave electromagnetic simulations qualitatively and quantitatively characterize their near‐field focusing and efficient evanescent‐to‐propagating wave conversion. When fully embedded in a PDMS matrix, TBY microspheres enabled super‐resolution imaging of anodic aluminum oxide and other nanoscale samples, resolving features down to 50 nm and attaining a maximum magnification of ∼4.34× on 100 nm grating structures. We show that image‐plane selection and precise axial alignment critically influence image clarity, contrast, and magnification, and we systematically investigate these trade‐offs across sphere diameters. An ultramicroscopic objective (UO) module integrating a plano‐convex lens with an embedded microsphere was developed to provide micrometer‐precise positioning, reusability, and straightforward compatibility with commercial microscopes. The high near‐infrared transmittance, low dispersion, and thermal stability of fluorotellurite glass indicate promising applications in deep‐tissue near‐infrared super‐resolution, multi‐band spectroscopic nanoscopy, and laser micro‐machining.

## Graphical abstract

Microsphere lenses made from fluorotellurite glass with a high refractive index and excellent transparency were used to develop a super‐resolution imaging technique and ultramicroscopic objective modules. This approach enables efficient, stable, and precisely controllable imaging with broad potential in life sciences and nanotechnology.

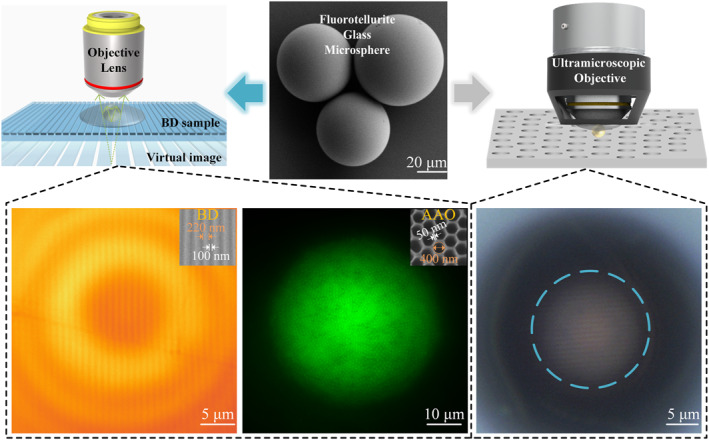

## 1 Introduction

Optical microscopy is an indispensable tool in scientific research due to its non‐destructive nature and capacity for real‐time imaging. It plays a critical role across numerous fields, including biology, materials science, and nanotechnology. As the frontiers of knowledge expand, the focus of investigation has progressively shifted from the macroscopic and microscales down to the nanoscale and beyond. However, the resolution of conventional optical microscopy is constrained by the diffraction limit, a fundamental barrier where high‐frequency spatial information is lost in the far field. This limit, defined by Abbe's equation as *d* = 0.5*λ*/NA, where *λ* is the wavelength of the illumination light and NA is the numerical aperture of the objective lens, restricts the minimum resolvable feature size [1]. The persistent demand for higher resolution drives the development of technologies capable of surpassing this limitation.

Consequently, several advanced optical nanoscopy techniques have been extensively developed. These methods, such as stimulated emission depletion microscopy, photoactivated localization microscopy, stochastic optical reconstruction microscopy, and scanning near‐field optical microscopy, among others, can achieve resolution beyond the diffraction limit [2, 3, 4, 5, 6, 7, 8, 9]. Despite their success, many of these approaches face challenges including low imaging speeds, potential sample damage from fluorescence labeling, and reliance on complex bulky equipment. In contrast, dielectric microspheres represent a unique class of superlens that can enable label‐free and real‐time super‐resolution imaging. These microspheres function by converting the evanescent near‐field waves, which carry high‐resolution information into propagating waves that can be detected by a standard microscope, producing a virtual image with sub‐diffraction‐limited detail [10, 11, 12]. This technique is not only cost‐effective and label‐free, but also readily integrable into existing optical microscope systems, showing great potential for biological imaging [13], semiconductor inspection [14] and the visualization of nanoscale manipulation [15].

Wang et al. first demonstrated that silica microspheres placed directly on a sample could resolve sub‐diffraction‐limited nanostructures under a conventional optical microscope [16]. Subsequent research further explored this principle, for instance, Hao et al. used silica microspheres partially immersed in anhydrous ethanol to image Blu‐ray discs with enhanced contrast [17], whereas other studies showed that liquid microlenses could also achieve super‐resolution [18]. An advancement was reported by Darafsheh et al., who utilized high‐refractive‐index (*n* > 1.7) barium titanate glass (BTG) microspheres to resolve sample features as small as one‐seventh of the illumination wavelength. Their findings indicate that microsphere‐assisted super‐resolution imaging not only surpasses the diffraction limit to enable label‐free imaging but also serve as a valuable tool for the observation and analysis of biological samples [19]. Compared to low‐refractive‐index counterparts, high‐refractive‐index microspheres generally provide higher magnification, enhanced resolution, and a larger field of view (FoV). Furthermore, their typical requirement for immersion in a liquid medium makes them naturally suited for biological application in various solutions [20, 21, 22, 23].

Given these advantages, high‐refractive‐index microspheres constitute a core research focus in this field. BTG microspheres are currently the most widely used, though other materials such as titanium dioxide and chalcogenide glass have also been explored [24, 25]. However, microspheres made from these existing high‐index materials often exhibit certain limitations. For instance, BTG microspheres may have limited optical transparency in mid‐infrared spectral ranges. Titanium dioxide microspheres, while offering exceptionally high refractive indices, typically require complex manufacturing processes and can introduce possible optical scattering and interfacial losses, thereby compromising high‐quality imaging. Chalcogenide glass microspheres, on the other hand, often suffer from strong light absorption in the visible region, and their constituent elements may pose potential toxicity concerns. Collectively, these factors hinder the simultaneous realization of high refractive index, high optical transparency, and excellent material stability, limiting their broader application.

To address these challenges, we developed fluorotellurite glass microspheres with a composition of TeO_2_–BaF_2_–Y_2_O_3_ (TBY) using a high‐temperature floating‐zone melting technique. This preparation technique has also been used in the fabrication of microspheres from materials such as fluoride glass, phosphate glass, and tellurite glass [26, 27, 28]. The resulting microspheres, with diameters ranging from 10 to 200 μm, exhibit a high refractive index of approximately 1.9 and a visible‐light transmittance of up to 85% (See Supporting Information S1: Figure S1) [29]. Thanks to these materials‐level advantages, TBY glass provides a well‐balanced combination of a relatively high refractive index, high visible‐light transmittance, potential near‐infrared transparency, and good thermal stability.

The combination of high transmittance and high refractive index minimizes optical loss while enhancing near‐field focusing and the conversion of evanescent waves into propagating waves, thereby allowing the extraction of more high frequency spatial information from the sample for improved resolution. In this work, we fully embedded the TBY microspheres in a polydimethylsiloxane (PDMS) matrix, leveraging its biocompatibility and chemical stability [30], to achieve super‐resolution imaging of anodic aluminum oxide (AAO) samples with feature sizes as small as 50 nm. Imaging tests on Blu‐ray disc (BD) gratings, demonstrated a maximum magnification of ∼4.34X.

Furthermore, we developed a dedicated objective lens adaptor to integrate the TBY microspheres with commercial microscope objectives, forming an ultramicroscopic objective (UO) module. This design allows for flexible integration with various optical microscope platforms, enabling precise localized super‐resolution imaging. The module facilitates the reusable application of microsphere lenses with stable performance, offering flexible spatial adjustability and micrometer‐scale positioning. This system supports the repeated imaging of different specimens, significantly enhancing imaging efficiency and broadening its applicability across diverse scenarios. The TBY microsphere‐based technique and UO module are capable of visualizing nanoscale structures down to several tens of nanometers. Finally, the favorable properties of fluorotellurite glass, such as its high near‐infrared transmittance, low dispersion, and excellent thermal stability, suggest its potential for future applications in deep‐tissue near‐infrared super‐resolution imaging [31], multi‐band spectroscopic nanoscopy [32], and high‐power laser micro‐machining [33].

## 2 Materials and Methods

### 2.1 Imaging and Characterization Equipment

Super‐resolution imaging experiments were conducted using an upright optical microscope (ZEISS Axio Imager. M2p) equipped with a mercury lamp light source (ZEISS HAL 100, central wavelength *λ* ≈ 616 nm) and a camera (ZEISS Axiocam 506 mono). No intentional adjustment of the illumination angle or polarization was applied, resulting in quasi‐normal‐incidence broadband illumination. Correspondingly, the simulations employed a normally incident plane wave at a wavelength of 616 nm as a representative excitation condition. The imaging tests utilized three different objective lenses: a 100X oil‐immersion objective (ZEISS EC Plan‐NEOFLUAR, NA = 1.3), a 63X water‐immersion objective (ZEISS W Plan‐APOCHROMAT, NA = 1.0), and a 50X air objective (Olympus LMPLanFL N, NA = 0.5). For comparative analysis and to verify nanoscale features, sample morphology was characterized using a Zeiss EVO MA10 scanning electron microscope.

### 2.2 Preparation of Fluorotellurite Glass Microspheres

The fabrication process for the TBY glass microspheres is illustrated in Supporting Information S1: Figure S2. The procedure began with the synthesis of bulk TBY glass in a muffle furnace. The resulting glass blocks were subsequently crushed and finely ground using an agate mortar. The ground material was then sieved to separate the powder into distinct particle size fractions. These size‐classified powders were gradually introduced into the feeding zone of a vertical furnace, which consists of three sections: a feeding zone, a heating zone, and a collection zone. As the powder entered the heating zone, the high temperature caused it to melt. Driven by surface tension and gravity, the molten droplets spontaneously formed into microspheres. These microspheres then descended into a water‐cooled quartz tube within the collection zone, where they were collected and stored in vials for future use.

### 2.3 Fabrication of Microsphere‐Assisted Imaging Samples and Ultramicroscopic Objective

To prepare samples for microscope‐assisted imaging, TBY microspheres stored in anhydrous ethanol were first dispersed onto the sample surface. The ethanol was then allowed to evaporate, leaving the microsphere in place. Subsequently, a PDMS solution (Sylgard 184, Dow Corning, *n* ≈ 1.41) was prepared by mixing the base with a curing agent at a 10:1 mass ratio and degassing it under vacuum to remove air bubbles. This PDMS solution was carefully applied to cover the sample and microspheres using a needle tip. Finally, the assembly was cured in an oven at 90°C for 20 min to produce the final imaging specimen.

The assembly of the UO module began by attaching a fixed coupler to the host objective at a predetermined position. Using a three‐axis adjustment stage and a tapered fiber, a small droplet of UV‐curable adhesive was deposited onto the center of a quartz glass substrate. A custom fabricated TBY microsphere lens was then gently transferred onto the adhesive droplet, achieving partial immersion. The adhesive was cured by exposure to UV light for 30 min, resulting in an integrated plano‐convex lens structure comprising the microsphere and the substrate. This configuration ensures that during operation, the microsphere contacts the sample before the glass substrate does. Finally, the assembled plano‐convex lens was bonded to a rotating sleeve. The rotation pitch of the sleeve was finely adjusted, according to the microsphere's diameter, to achieve precise alignment with the focal plane of the objective lens.

## 3 Results and Discussion

### 3.1 Principle and Simulations of Fluorotellurite Glass Microsphere‐Assisted Nanoscopic Imaging

**FIGURE 1:**
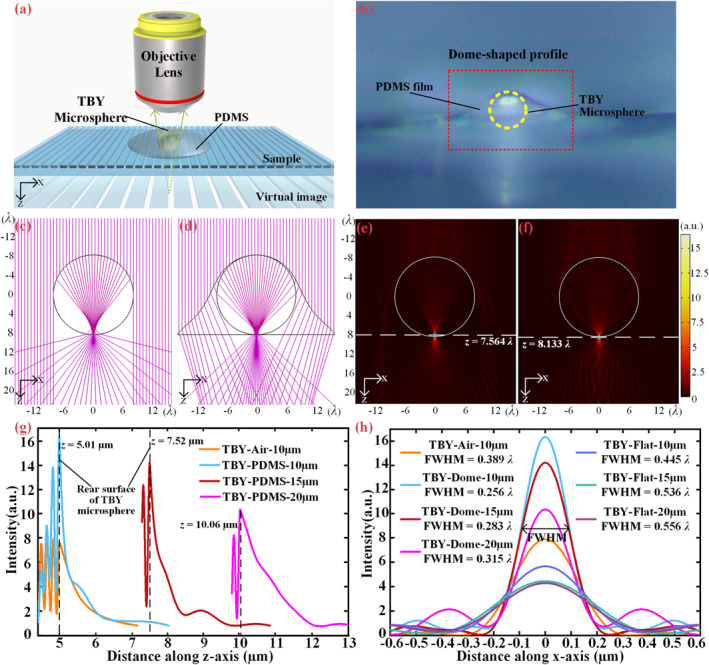
Theoretical mechanism analysis of TBY microsphere lens‐assisted super‐resolution imaging. (a) Schematic diagram of a reflection‐mode nanoscope based on a TBY microsphere fully embedded in a PDMS thin film. (b) Side‐view microscopic image of TBY microspheres embedded in the PDMS film. (c–d) Ray‐tracing simulations showing the refraction and convergence of vertically incident light through a TBY microsphere (c) in air and (d) fully immersed in PDMS. (e–f) Electric field intensity distributions obtained from full‐wave simulations under (e) air and (f) PDMS environments, corresponding to fully immersed conditions. (g) Rescaled intensity profiles along the *z*‐axis for microspheres of various diameters in air and dome‐shaped PDMS media, respectively. (h) Corresponding FWHM at the position of maximum light intensity in the PNJ field distributions for microspheres of different diameters under different immersion conditions.

Figure 1a illustrates a schematic of the nanoscopic imaging principle using a TBY microsphere fully embedded within a PDMS elastic layer. To access the evanescent waves that carry sub‐diffraction‐limited spatial information and decay exponentially across the interface—near the surface of nanostructures, the microsphere is placed in direct contact with the nanostructures. Under illumination from a reflection‐mode bright‐field optical microscope, the microsphere generates a magnified virtual image of these fine features beneath it, which is then captured by the objective lens. The PDMS encapsulation layer conforms to the microsphere's shape, bulging outward to form a dome‐like structure that extends slightly above the microsphere's apex. A magnified side‐view of this PDMS profile is shown in Figure 1b, where the contour of the encapsulation layer is visible due to the divergence of reflected light from its sloped surface. The position of the embedded TBY microsphere is indicated by a yellow dashed circle.

When the microsphere's size is comparable to the wavelength of the incident light, the principles of ray optics become insufficient as they neglect diffraction and interference. In this regime, the interaction of light with the microsphere is more accurately described by Mie scattering theory, which is based on wave optics [34]. Although ray optics cannot provide quantitative predictions of image characteristics at this scale, it remains a valuable qualitative tool for analyzing imaging behavior by approximating light propagation with idealized ray trajectories and neglecting diffraction effects [35]. Therefore, we conducted both ray‐tracing simulations and full‐wave numerical simulations using the finite element method to theoretically analyze the imaging performance of the TBY microsphere. These approaches provide qualitative and quantitative insights, respectively. For simplicity, a two‐dimensional circular structure was used to model the three‐dimensional sphere.

In the ray optics analysis (Case 1), Figure 1c illustrates the convergence of light rays after a plane wave passes through the immersion‐type TBY microsphere in an air environment. Here, 50 incident rays are emitted downward along the *z*‐axis from the top at *z* = −15.87*λ*. Upon reaching the interface between air and the TBY microsphere, the rays refract into the microsphere. Among them, 23 rays bent toward the sphere's center and converged within it. The region of highest ray density, indicating the primary focus, is located between the geometric center and the rear surface of the sphere. After this convergence, the rays diverge outward. A substantial portion of the light deviates markedly from the *z*‐axis centerline, diverging at a wide angle within the sphere. When the surrounding medium is changed to PDMS, with a cladding layer conformally adhered to the microsphere, the ray behavior changes significantly, as shown in Figure 1d. Here, 27 out of the 50 incident rays are captured and refracted into the microsphere. These rays exit the microsphere at smaller angles and diverge more gradually compared to the air environment. The focal region also shifts closer to the vicinity of the rear surface of the microsphere. It is noteworthy that even rays bypassing the microsphere exhibit a tendency to converge toward the optical axis after traversing the PDMS film, a phenomenon that may partly explain how some irregular transparent media can achieve super‐resolution imaging in the absence of microsphere lenses.

For the wave optics analysis (Case 2), full‐wave simulations were conducted to investigate the light field intensity distribution for a 10 μm‐diameter microsphere under 616 nm plane wave illumination in both air and PDMS. As shown in Figure 1e, when in air, the incident wave is refracted by the microsphere and focuses to a point (at the blue dot) of maximum intensity located approximately *z* = 7.564*λ* inside the sphere. In contrast, when immersed in PDMS (Figure 1f), the focal spot shifts downward to the vicinity of the contact point between the microsphere and the sample, forming a pronounced photonic nanojet (PNJ)‐like distribution, which then diverges outward, as illustrated in Figure 1f. A comparison between the geometrical and wave optics simulations shows strong consistency. In air, both the light convergence point and the maximum intensity region are located inside the microsphere, preventing the formation of a PNJ and the conversion of near‐field evanescent waves into propagating waves, which is unsuitable for far‐field super‐resolution imaging. In the PDMS environment, however, both the light convergence points and the maximum intensity spot shift to the rear surface of the microsphere, enabling PNJ formation and the magnification of subwavelength details, which are necessary conditions for super‐resolution. The reduced divergence angle observed in the ray‐tracing results is also apparent here, with outgoing waves converging slightly toward the central *z*‐axis (below the white dashed line), which may contribute to image enhancement. Furthermore, the geometric morphology of the PDMS coating influences the spatial characteristics and field distribution of the PNJ; simulation results for different PDMS surface morphologies are presented in Supporting Information S1: Figure S3. Moreover, the illumination conditions can influence the detailed characteristics of PNJ formation and the resulting image contrast. Parameters such as wavelength, incidence angle, coherence, and polarization may modify the local field distribution near the microsphere‐sample interface, thereby affecting the efficiency of evanescent wave coupling.

Figure 1g illustrates the focal positions and intensity distributions along the *z*‐axis for different background media (air and PDMS) and microsphere diameters (10 μm, 15 and 20 μm). Microspheres immersed in dome‐shaped PDMS exhibit a significantly enhanced peak focal intensity compared with those in air.

Furthermore, smaller microspheres (e.g., 10 μm in diameter) demonstrate higher efficiency in concentrating captured light, resulting in a stronger focusing intensity [36]. The full width at half maximum (FWHM) of the PNJ is a key parameter for evaluating imaging resolution. Figure 1h compares the PNJ FWHM under different conditions. The results indicate that a 10 μm microsphere in a dome‐shaped PDMS medium generates a PNJ with the narrowest waist of approximately 158 nm (∼0.256*λ*), demonstrating excellent focusing capability. In addition, microspheres embedded in dome‐shaped PDMS produce a significantly narrower PNJ waist than those embedded in flat PDMS at the same diameter.

### 3.2 Super‐Resolution Imaging Performance of Fluorotellurite Glass Microsphere‐Assisted Nanoscopy

**FIGURE 2:**
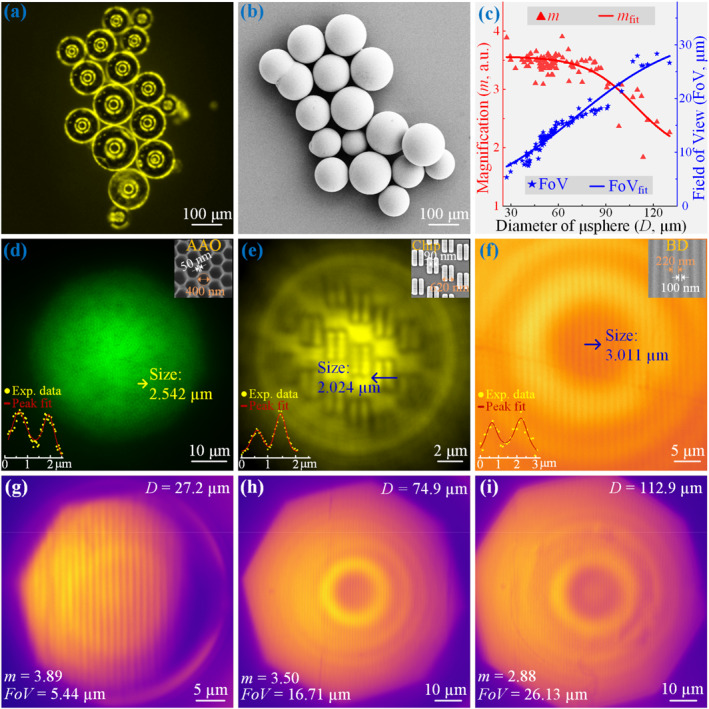
Super‐resolution imaging results of TBY microsphere‐assisted nanoscopy. (a) Optical microscopy image of TBY microspheres prepared by high‐temperature floating zone melting method, captured under a 5X objective lens. (b) SEM image showing the morphological features of the TBY microspheres. (c) Relationship between the magnification factor (*m*) and field of view (FoV) as a function of TBY microsphere diameter (27–130 μm) during super‐resolution imaging of the BD sample. (d–f) Magnified virtual images of (d) an AAO structure, (e) a semiconductor chip circuit structure, and (f) a BD structure, generated by TBY microspheres with diameters of 62 μm, 14 μm, and 55 μm, respectively. The corresponding sample parameters are as follows: AAO, period 450 nm (50 nm spacing and 400 nm pore size); chip circuit, period of 620 nm (265 nm linewidth and 90 nm gap); and BD, period of 320 nm (220 nm linewidth and 100 nm spacing). The line‐cut arrow indicates the direction used for extracting the intensity profile and the corresponding pixel size. In each inset: the lower left corner shows the intensity profile with its fitted curve, whereas the upper right corner displays the corresponding SEM image. (g–i) Magnified virtual images of BD grating structures obtained using TBY microspheres with diameters of 27.2 μm, 74.9 μm, and 112.9 μm, respectively. The corresponding magnifications and FoV values, indicated in the lower left corner of each image, are 3.89X and 5.44 μm, 3.50X and 16.71 μm, and 2.88X and 26.13 μm.

The high‐temperature floating zone melting method is well‐suited for processing oxide glass materials, allowing for the efficient production of thousands of high‐quality microspheres in a single batch with excellent repeatability and sphericity. Figure 2a,b show the optical microscopy image and SEM image of the fabricated TBY microspheres, respectively. These images confirm that the majority of the microspheres possess excellent sphericity and a smooth surface, with only a minor fraction exhibiting slight defects. The high surface smoothness is critical as it minimizes light scattering and energy loss, thereby establishing a solid foundation for high‐quality super‐resolution imaging.

The magnification factor *m* is defined as the ratio of the distance between two points in one period of the virtual image to the corresponding distance on the actual sample. Figure 2c presents the relationship between magnification (*m*), field of view (FoV), and microsphere diameter (ranging from 27 to 130 μm) during the imaging of a commercial BD sample. This BD sample contains periodic grating structures with a minimum feature size of 100 nm, which cannot be resolved directly by a conventional optical microscope, as confirmed by the image taken with a 100X oil‐immersion objective lens shown in Supporting Information S1: Figure S5a. The data in Figure 2c reveal a negative correlation between microsphere diameter and magnification *m* (red curve), and a positive correlation between diameter and FoV (blue curve).

As the diameter increases from 30 to 80 μm, the magnification *m* decreases slightly from ∼4X to 3.5X. A further increase in diameter to the 80–130 μm range causes the magnification to drop from 3.5X to 2.3X. Meanwhile, the FoV expands with increasing diameter. The slope of the FoV increase is steeper in the 30–80 μm range, indicating a rapid expansion of the observable area. For diameters larger than 80 μm, however, the imaging quality deteriorates, and only the central region of the microsphere can resolve the BD gratings clearly, resulting in a slower rate of FoV growth.

The imaging performance was further evaluated using TBY microspheres fully embedded in PDMS on three distinct sample types: nanopore arrays in anodized aluminum oxide (AAO), circuit structures on a semiconductor chip, and grating structures on a BD. In the magnified virtual image, the minimum discernible gap in the sample is visually determined as a criterion for evaluating imaging performance. This criterion reflects the discernibility of the image, and therefore provides an upper bound of the system's effective resolution, rather than the true optical resolution. To further quantify the effective imaging resolution, we performed a simple PSF‐based analysis (see Supporting Information S1: Figure S4). The super‐resolution imaging results for these samples are presented in Figure 2d–f. The inset in the upper right corner of each panel shows the corresponding SEM image of the sample, whereas the lower left inset illustrates the intensity profile and its fitted curve along the direction indicated by the arrow in the main image. The corresponding pixel size is labeled at the position indicated by the arrow. The AAO sample featured a periodicity of 450 nm and a pore diameter of ∼400 nm, with a minimum feature size of 50 nm in the gaps between adjacent pores (see the microscopic image in Supporting Information S1: Figure S5b). Using a 62 μm‐diameter TBY microsphere, a clear virtual image of the AAO nanostructures was obtained, with a calculated magnification of 3.01X.

The semiconductor chip circuit structure consists of two adjacent stripes with a center‐to‐center spacing of 355 nm and an inter‐stripe trench width of 90 nm. When imaged directly with a 100X objective lens, the 90 nm trench cannot be resolved, resulting in an image with low contrast, as shown in Supporting Information S1: FigureS5c. In contrast, the virtual image obtained using a 14 μm TBY microsphere, presented in Figure 2e, is significantly clearer and successfully resolves this sub‐diffraction‐limit structure. The intensity profile and fitted curve in the inset yield a magnification factor of approximately 3.46X. Similarly, for the BD sample with a grating period of 320 nm (220 nm stripe width and 100 nm spacing), a clear and magnified virtual image was obtained using a 55 μm TBY microsphere, as shown in Figure 2f. The imaging quality is optimal at the center of the microsphere, the intensity profile analysis indicated a maximum magnification of 4.34X. Figure 2g–i present BD imaging using microspheres of different diameters, illustrating the corresponding trends in microsphere diameter, *m*, and FoV.

It is noteworthy that the BD surface is coated with a highly‐reflective metallic layer. Light reflected from this layer undergoes multiple interference with light reflected from the microsphere‐sample interface, producing concentric Newton's rings. The spacing of these rings is determined by the optical path difference [37]. Pronounced Newton's ring patterns are observed in the experimentally obtained virtual images. However, if the protective layer on the BD surface is removed to expose the grating structure, slight damage to the data layer may occur, potentially destabilizing these interference patterns.

Based on geometric optics ray tracing, the magnification in microsphere‐assisted imaging can be approximated by the formula $m=n^{\prime }/|2-n^{\prime }|$, where $n^{\prime }=n_{p}/n_{\text{bg}}$ (with *n*_p_ and *n*_bg_ representing the refractive indices of the microparticle and the surrounding background medium, respectively). According to this formula, the theoretical magnification for a TBY microsphere immersed in PDMS is approximately *m* ≈ 2.06. In practice, however, effects such as enhanced interference [38], localized surface plasmon resonance [39], and sample‐specific structural characteristics can contribute to a further enhancement of the magnification, leading to the observed maximum values that exceed the theoretical prediction.

### 3.3 Comparative Analysis of Imaging at Different Image Planes

The pronounced depth‐of‐field behavior in microsphere‐assisted super‐resolution imaging warrants a detailed investigation. Our experiments revealed that when the objective lens is focused on the equatorial plane (the central plane) of the microsphere, a clear outline of the microsphere itself is obtained, but the sub‐diffraction‐limit features on the sample remain unresolved. As the focal plane is gradually adjusted closer to the sample surface, virtual images of the nanoscale structures, formed with the assistance of the microsphere, begin to emerge. With continued fine‐tuning, high‐contrast, and high‐resolution super‐resolved images are achieved. The focal plane must be moved downward from the microsphere's center by a distance greater than its radius by several wavelengths (*λ*) before reaching the optimal imaging region near the microsphere's rear surface, where the best image quality is obtained.

**FIGURE 3:**
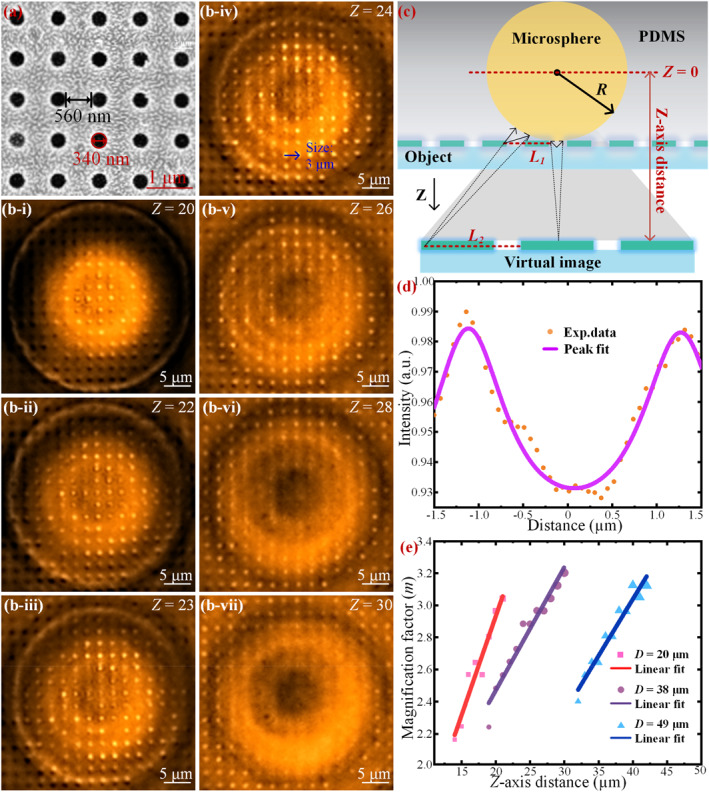
Comparison of imaging performance at different image planes with the assistance of TBY microspheres. (a) SEM image of the dot array structure on the chip. The diameter of each dot is 340 nm, and the center‐to‐center spacing between adjacent dots is 560 nm. (b‐i) to (b‐vii) Virtual images of the dot array captured at *Z* = 20–30 μm using a 38 μm‐diameter TBY microsphere lens. The images illustrate how changes in the image plane affect image quality. (c) Schematic illustration of image variation achieved by adjusting the objective's focal plane along the *Z*‐axis via stage movement. *R*: radius of the microsphere, *L*_1_: actual distance between two points on the microstructure of the sample, *L*_2_: distance between the corresponding two points in the virtual image. (d) Intensity profile and its fitting curve corresponding to the blue arrow marked in (b‐iv). (e) Relationship between the *Z*‐axis position and magnification (*m*) under imaging assisted by TBY microspheres with diameters of 20, 38, and 49 μm, along with corresponding linear fitting.

To study this phenomenon more intuitively and quantitatively, we employed a sample chip with a periodic circular dot array. The SEM image in Figure 3a shows that the array has a dot‐to‐dot spacing of 560 nm, with each dot measuring 340 nm in diameter. For high‐precision imaging, the prepared sample was placed on a microscope stage with three‐dimensional fine‐tuning capabilities. Imaging and focusing were controlled using ZEISS ZEN Blue software with a 63X objective lens. Figure 3c provides a schematic of the microsphere‐assisted imaging process for the dot array, illustrating the adjustment of the relative position along the *Z*‐axis. The equatorial plane of the microsphere is defined as the origin (*Z* = 0), with the positive *Z*‐direction extending toward the sample surface. The distance from the microsphere's center to the sample surface is equal to its radius *R*. Since the objective's focal length is fixed, raising the sample stage is equivalent to lowering the focal plane. The distance between the equatorial plane and this image plane is defined as the *Z*‐direction focal offset. The *m* is the ratio of the distance between features in the virtual image (*L*_2_) to their actual distance (*L*_1_), that is, *m* = *L*_2_/*L*_1_.

Figure 3b presents the imaging results of the dot‐array structures using a 38 μm‐diameter TBY microsphere at different image planes along the *Z*‐axis. The images in Figure 3b‐i to b‐vii correspond to focal depths incrementally adjusted from 20 to 30 μm. As the image plane shifts downward (increasing *Z* value), the quality of the virtual image first improves and then deteriorates, confirming the existence of an optimal image plane where contrast and clarity are maximized. Specifically, the image acquired at *Z* = 24 μm (Figure 3b‐iv) demonstrates the optimal clarity and optical contrast. Concurrently, the FoV expands outward from the optical axis center, and the magnification increases linearly with *Z*. The trend in FoV change is closely linked to image contrast: beyond the optimal focal depth (e.g., at *Z* = 28 and 30 μm), the central region of the image becomes blurred, and further expansion can impair the accurate visualization of structural details, thereby degrading overall imaging performance.

For the optimal image at *Z* = 24 μm, the intensity profile across two adjacent dot centers (connected by a blue arrow) was extracted and analyzed, as shown in Figure 3d. The profile exhibits a symmetric peak‐valley structure within the range of −1.5–1.5 μm, with the peak positions corresponding to the dot centers. The measured distance between peaks (*L*_2_) is 2.45 μm, yielding a calculated magnification factor of *m* = 2.72. Figure 3e further illustrates the relationship between magnification and the vertical distance (*Z*) for TBY microspheres with three different diameters (*D* = 20 μm, 38 μm, and 49 μm). A strong positive correlation is observed between *Z* and *m* for all microsphere sizes. Moreover, smaller‐diameter microspheres achieve higher magnifications at their respective optimal imaging positions, a finding that is consistent with previous studies.

### 3.4 Ultramicroscopic Objective Based on Fluorotellurite Glass Microsphere

In conventional microsphere‐assisted super‐resolution imaging, high‐refractive‐index microspheres are typically immersed in liquid or embedded within flexible films such as PDMS. While these approaches have successfully demonstrated imaging beyond the diffraction limit, they remain constrained by limited spatial control, poor reusability of the microsphere, and reduced adaptability for dynamic imaging scenarios. A primary limitation is the random deposition of microspheres on the sample, which offers low positional precision and makes it difficult to target and repeatedly image specific regions of interest.

To address these challenges, we developed a modular ultramicroscopic objective (UO) that integrates a microsphere lens with custom mounting connectors and a standard microscope objective [40, 41, 42, 43, 44]. This configuration allows for the precise positioning of the microsphere, enabling targeted imaging across extended sample areas. Moreover, the separable design, which decouples the microsphere assembly from the sample itself, ensures that the self‐assembled plano‐convex microsphere lens remains intact and reusable over multiple imaging sessions. The UO module thus combines high‐resolution performance with reusability, precise spatial control, and scannable flexibility, offering a more versatile and robust platform for nanoscale optical imaging.

**FIGURE 4:**
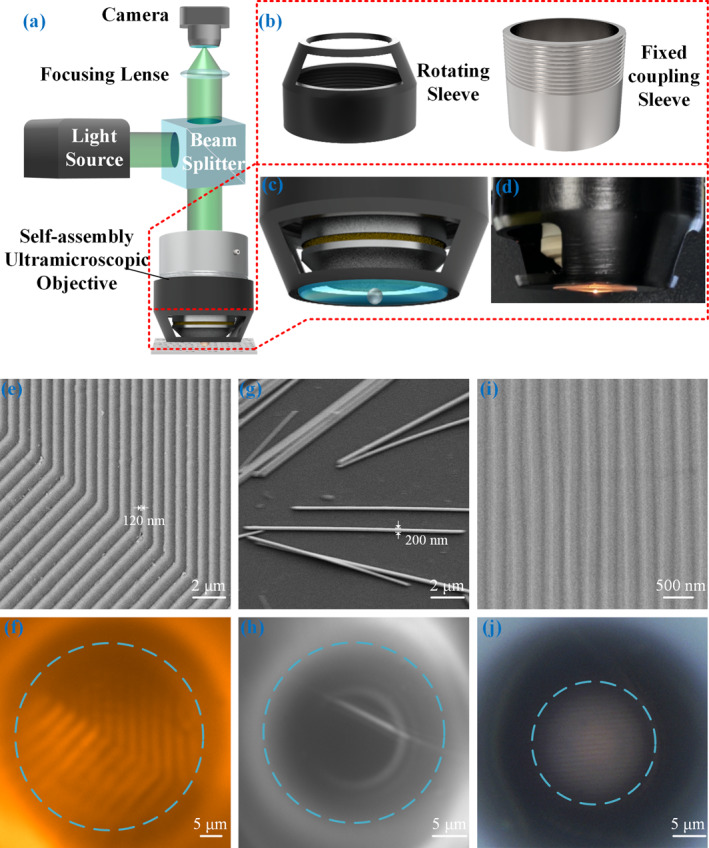
TBY microspheres‐assisted ultramicroscopic objective and its application in super‐resolution imaging. (a) Schematic diagram of the optical path of the MAM with an integrated UO. (b) Structural diagram of the rotating coupler and the fixed coupler components used for UO. (c) Schematic and (d) physical images of the UO. (e–j) SEM images of various samples and their corresponding magnified images acquired with the assistance of the UO. (e) SEM image of striped structures on a chip circuit with a period of 800 nm and a gap of 120 nm between adjacent stripes; (f) Magnified image of the striped structures captured using a Zeiss 63X water immersion objective assisted by UO. (g) SEM image of a silver nanowire with a diameter of ∼200 nm. (h) Magnified image of the silver nanowire captured using a Zeiss 63X water immersion objective with UO assistance. (i) SEM image of the BD structure. (j) Magnified image of the BD captured using an Olympus 50X air objective with UO assistance.

The optical path of the microsphere‐assisted microscopy (MAM) system equipped with the UO is illustrated in Figure 4a. White light from the source is reflected by a beam splitter into the objective lens, passes through the microsphere to illuminate the sample surface, and is reflected back. The reflected light, now carrying fine structural details from the sample, travels back through the UO, passes through the beam splitter and a focusing lens, and is finally captured by the camera. Figure 4b shows an exploded view of the UO mounting structure, which consists of two main components: a rotating sleeve (left) for assembling and aligning the glass substrate, and a fixed coupler (right) that attaches to the objective lens via set screws. A magnified view of the fully assembled UO's bottom is presented in Figure 4c. The microsphere is precisely centered within the objective's FoV. The radius of curvature of the plano‐convex lens is determined by the volume of the UV‐curable adhesive droplet, its volumetric change during curing, and it’s wetting properties on the quartz substrate. A photograph of the operational UO is shown in Figure 4d, in which incident light converging through the quartz substrate forms a bright, intensified spot at the microsphere, indicating a strong focusing effect. This provides indirect evidence that the self‐assembled UO generates a PNJ‐like hotspot, confirming its excellent potential for super‐resolution imaging.

We experimentally evaluated the imaging performance and applicability of the assembled UO component. Using the same light source and a 63X water immersion objective as in previous setups, the theoretical diffraction‐limited resolution was 308 nm. To evaluate the UO's super‐resolution capability, we selected samples with feature sizes well below this limit. Figure 4e shows a circuit chip with striped structures titled at approximately 30° to the horizontal axis, where the gap between adjacent stripes is 120 nm. The super‐resolved image obtained with the UO, as shown in Figure 4f, clearly resolves these 120 nm gaps within the blue dashed circle, demonstrating excellent sub‐diffraction imaging. The UO enables precise localization on the sample, the FoV in this region is ∼249.3 μm^2^. Given that the TBY microsphere used has a diameter of 32.6 μm and equatorial cross‐sectional area of 837.1 μm^2^, this effective FoV covers about 29.8% of the area. A quantitative comparison between the UO and representative microsphere‐assisted imaging designs is summarized in Supporting Information S1: Table S1.

A second test was performed on a silver nanowire with a minimum lateral dimension of 200 nm, as shown in Figure 4g. The magnified image acquired using a UO with a 40.4 μm TBY microsphere is presented in Figure 4h, where the nanowire structure within the blue dashed circle is clearly identified. Here, the FoV is ∼674.6 μm^2^, accounting for about 52.6% of the microsphere's equatorial cross‐section area. Results from repeatability tests for super‐resolution imaging using the same UO are provided in Supporting Information S1: Figure S6.

To verify the UO's compatibility with different commercial microscope platforms, we designed a custom adapter for a 50X air objective on an Olympus microscope. Using this new UO component to image a BD sample (Figure 4i), we obtained the result shown in Figure 4j. The periodic grating stripes on the BD surface are clearly resolved within the microsphere's central FoV, which measures 194.2 μm^2^, or ∼25.7% of the microsphere's equatorial cross‐sectional area. Compared to traditional methods of immersing microspheres in liquids or elastomers, the TBY microsphere‐based UO shows a comparable or slightly improved effective FoV. Variations in the effective FoV among different UO units were observed, which may be attributed to slight differences in the adhesive droplet volume during assembly and microscopic geometrical variations of the cured plano‐convex lens.

## 4 Conclusions

In this study, we have presented a comprehensive investigation into the use of high‐refractive‐index fluorotellurite (TBY) glass microspheres for super‐resolution imaging, encompassing their fabrication, theoretical modeling, and experimental validation. The fabricated TBY microspheres demonstrated robust super‐resolution performance across diverse nanoscale samples, resolving feature sizes as small as 50 nm and achieving a maximum magnification of 4.34X. Our analysis revealed that the selection of the image plane is a critical parameter, with precise axial alignment being essential for optimizing image clarity and magnification. To assess the practical limitations of traditional methods, we developed an integrated ultramicroscopic objective (UO) module incorporating the TBY microsphere. This module provides precise spatial control, great reusability, and straightforward compatibility with standard commercial microscope systems. The inherent material properties of the fluorotellurite glass, including its high near‐infrared transmittance, low dispersion, and exceptional thermal stability, establish a strong foundation for extending this platform beyond visible light imaging. Consequently, this work paves the way for promising further applications in areas such as deep‐tissue super‐resolution imaging using near‐infrared light, multi‐band spectroscopic nanoscopy, and high‐power laser micromachining.

## Author Contributions

**Haonan Zhuo:** methodology, software, data curation, investigation, validation, formal analysis, visualization, writing – original draft. **Shengchuang Bai:** supervision, funding acquisition, project administration, resources. **Zhouyi Yu:** data curation, validation. **Zhenmin Wang:** data curation. **Zejie Zheng:** data curation. **Yu Zhuang:** data curation. **Yina Jiang:** data curation. **Tianyao Zhang:** data curation. **Hao Li:** validation, data curation. **Lixiang An:** validation, data curation. **Duanduan Wu:** project administration. **Xunsi Wang:** resources. **Hui Yang:** resources, funding acquisition, project administration, supervision, writing – review and editing. **Guoqiang Gu:** conceptualization, methodology, investigation, formal analysis, supervision, funding acquisition, visualization, project administration, resources, writing – review and editing.

## Funding

This work was financially supported by the National Natural Science Foundation of China (Nos. 62175252, 62205163 and 62475279), Guangdong Basic and Applied Basic Research Foundation (Nos. 2024A1515012320 and 2025A515012136), Natural Science Foundation of Ningbo (No. 2024J229), and China Postdoctoral Science Foundation (No. 2024M753396).

## Conflicts of Interest

The authors declare no conflicts of interest.

## Supporting information


Supporting Information S1

## Data Availability

The data that support the findings of this study are available from the corresponding author upon reasonable request.
