# Supplementary material for: Super‐Resolution Imaging With Fluorotellurite Glass Microspheres
Source: Nanophotonics. 2026 Feb 27;15(5):e70041. doi: 10.1002/nap2.70041 (PMC12964984; doi:10.1002/nap2.70041)
Supplement: Supplementary file 1 — Supporting Information S1 [file NAP2-15-e70041-s001.docx]

Supplementary Materials

Haonan Zhuo, Shengchuang Bai^*^, Zhouyi Yu, Zhenmin Wang, Zejie Zheng, Yu Zhuang, Yina Jiang, Tianyao Zhang, Lixiang An, Hao Li, Duanduan Wu, Xunsi Wang, Hui Yang^*^ and Guoqiang Gu^*,^[^^[[1]](#footnote-1)^^](#OLE_LINK139)

**Super-resolution Imaging with Fluorotellurite Glass Microspheres**


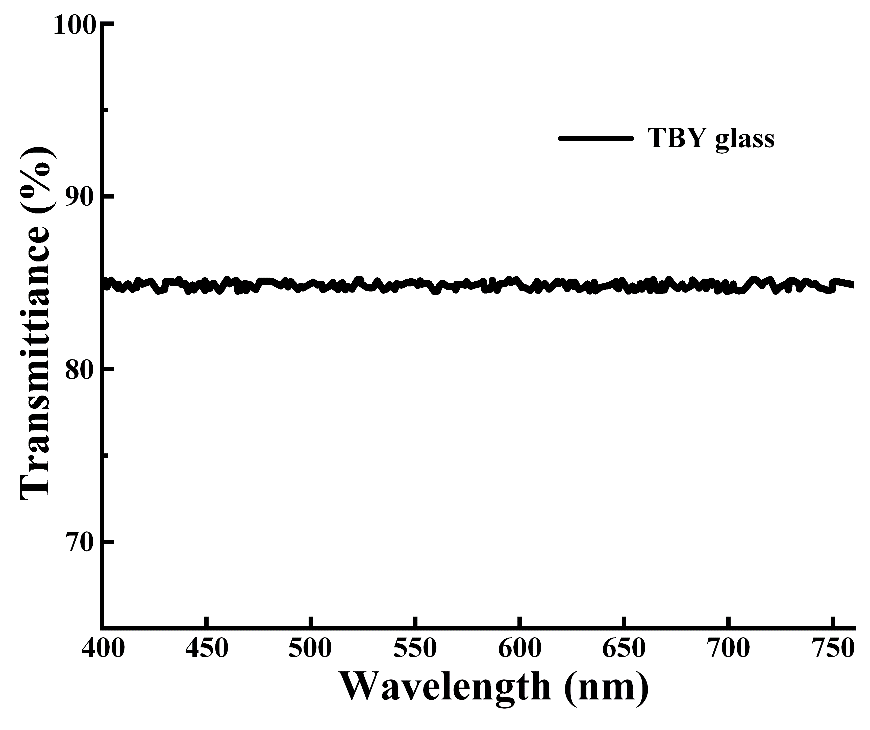


**Fig. S1:** Transmittance of TBY glass in the visible spectrum range of 400-760 nm.

**
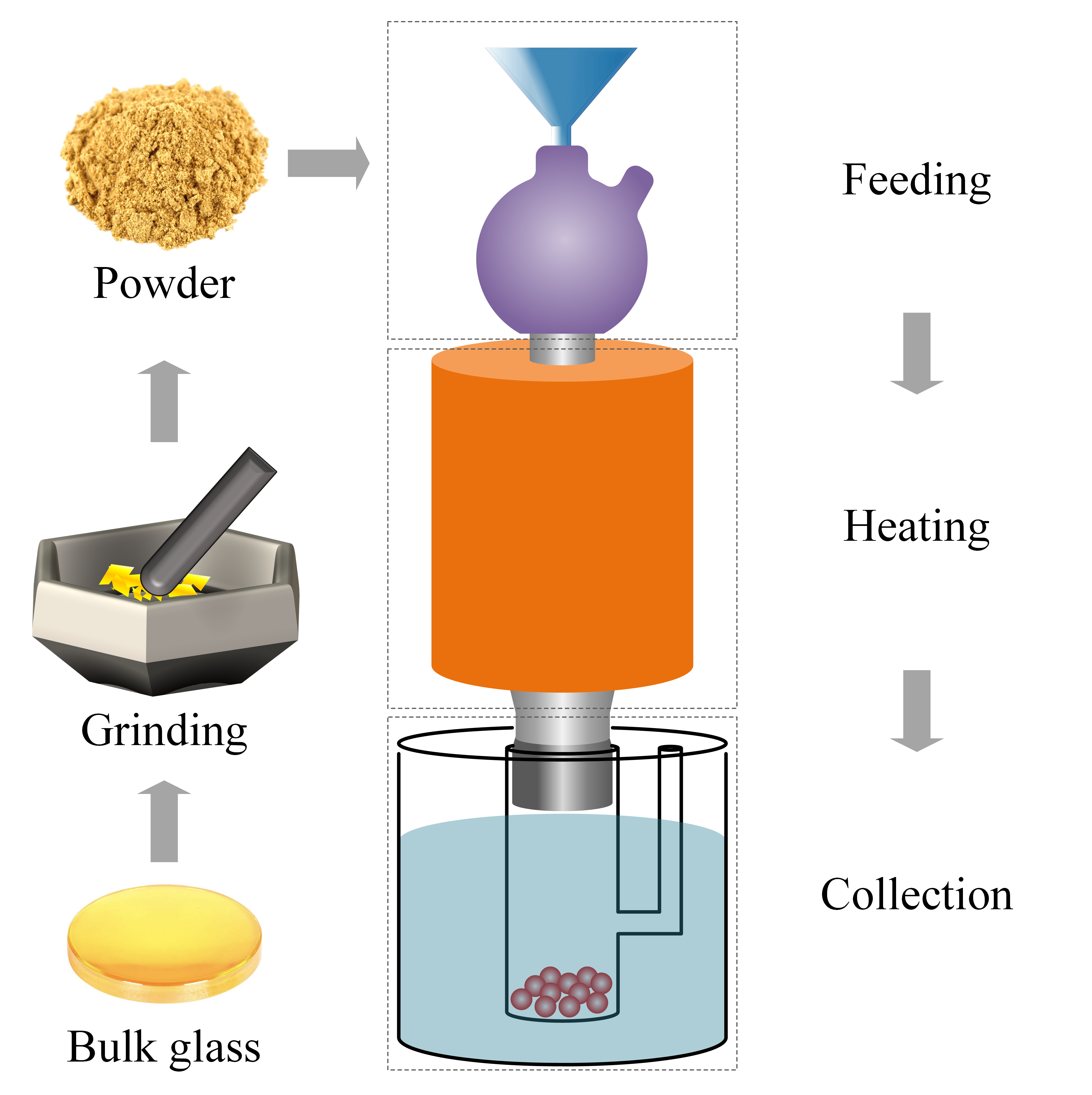
**

**Fig. S2:** Simplified flowchart of the high-temperature floating zone melting process, illustrating the main steps from raw powder preparation to melting microsphere formation, cooling and final collection.

In the experiments, both ray optics and wave optics simulations were performed to analyze the PNJ-like focusing characteristics of microspheres with different diameters (*D* = 15 µm and 20 µm), each coated with a dome-shaped PDMS films. A comparative study was also conducted between the outward-bulging coating configuration and the rectangular full-immersion configuration. Figs. S2(a) and S2(b) show ray-tracing simulations for 15-µm- and 20-µm-diameter TBY microspheres embedded in a dome-shaped PDMS, revealing that the focal region shifts toward the positive z-axis as the microsphere diameter increases. Corresponding full-wave simulations under identical conditions [Figs. S2(c) and S2(d)] exhibit similar outward PNJ-like focal shifts, with peak intensities located at *z =* 12.208*λ* (15 µm sphere) and *z* = 16.331*λ* (20 µm sphere). For a flat PDMS surface, full-wave simulations for microspheres with diameters ranging from 10-20 μm [Fig. S2(e-g)] indicate that the PNJ-like focus forms several wavelengths behind the rear surface and moves farther away with increasing sphere size. The dome-shaped coating produces a markedly stronger focusing intensity compared to the flat immersion configuration.


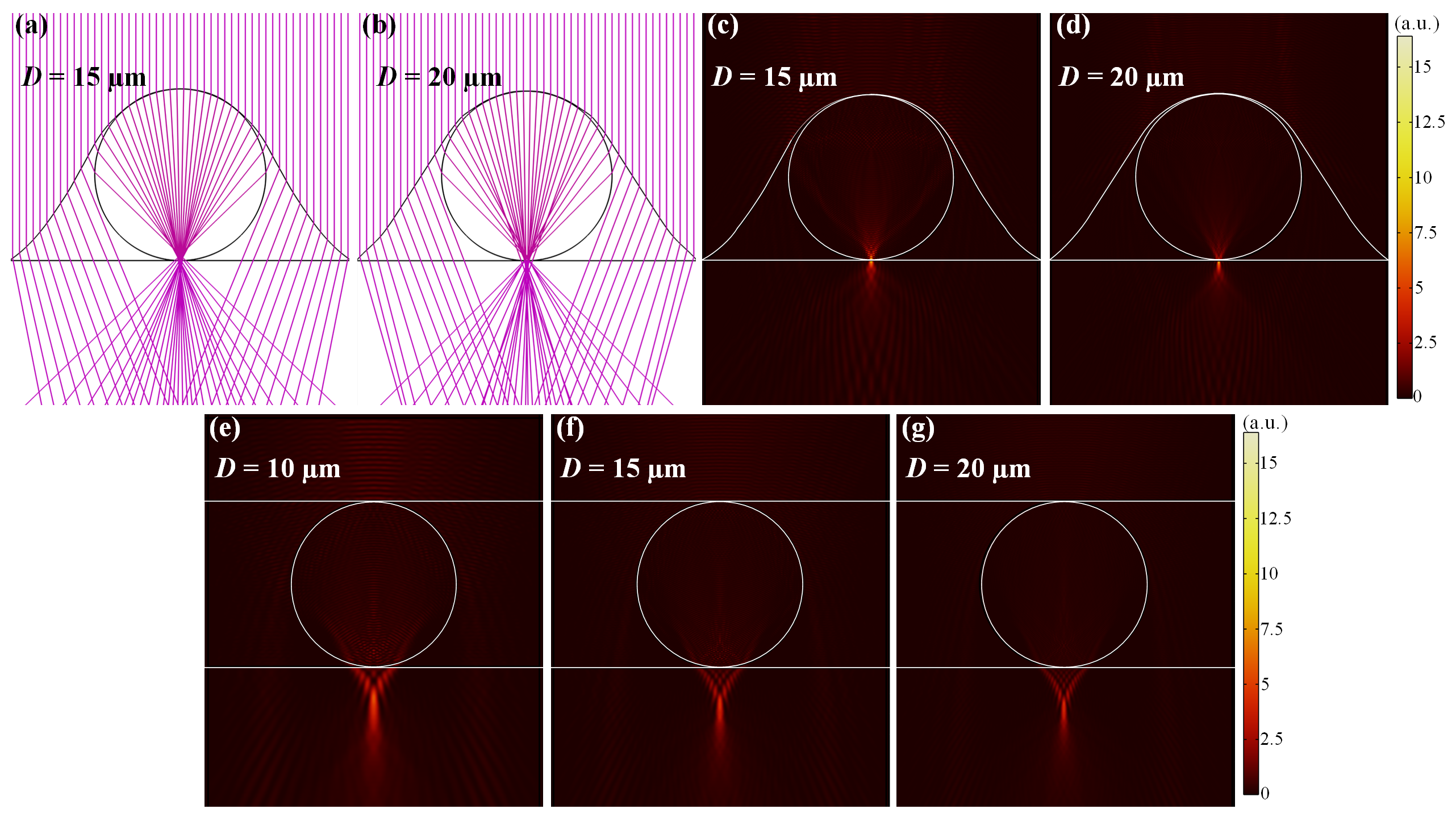


**Fig. S3:** (a-b) Ray tracing simulations based on geometrical optics for TBY microspheres with diameters of 15 µm and 20 µm under the outward-bulging PDMS coating configuration. (c-d) Full-wave simulations based on wave optics for the same microspheres under the same convex coating configuration. (e-g) Full-wave simulation results for TBY microspheres with diameters of 10 µm, 15 µm, and 20 µm under the rectangular full-immersion PDMS coating configuration.

The PSF describes the imaging system's response to an ideal point source and can be obtained by deconvolving the imaging function with the ideal sample structure function. Fig. S4 shows the convolution of the ideal AAO structure with PSFs of varying FWHMs: 40 nm, 70 nm, 100 nm, 120 nm, 150 nm and 200 nm. The PSF analysis indicates an effective resolution of approximately 200 nm. Since the AAO sample consists of finite-sized periodic structures rather than point sources, the PSF may not directly correspond to the resolution defined by the smallest recognizable feature.


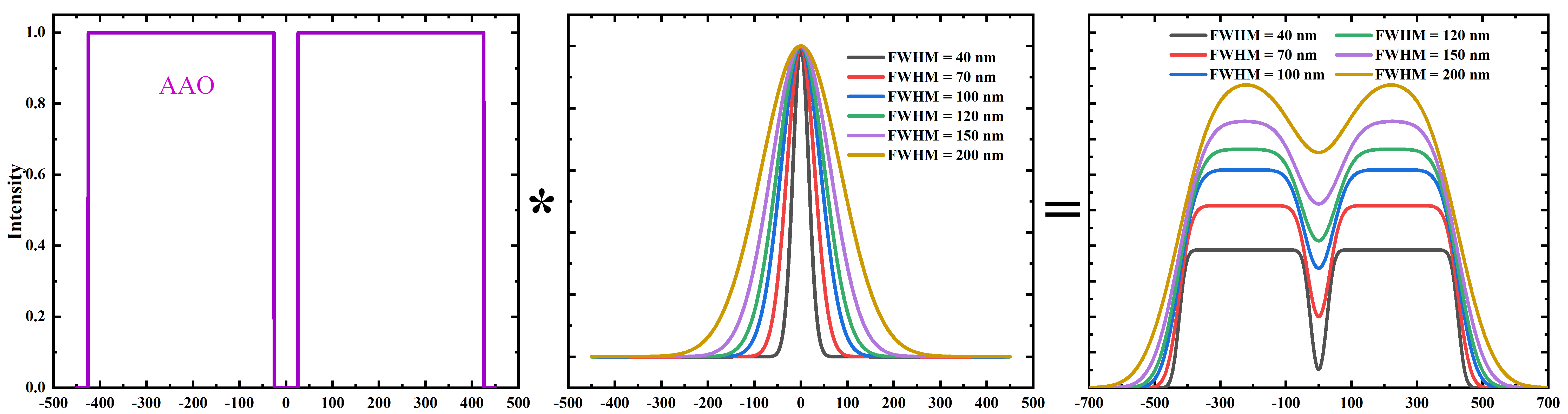


**Fig S4:** Convolution of ideal AAO structure with PSF of different FWHM.

Figs. S5(a–c) show BD, AAO, and chip circuit structures imaged using a 100× objective, where the minimum features remain unresolved.


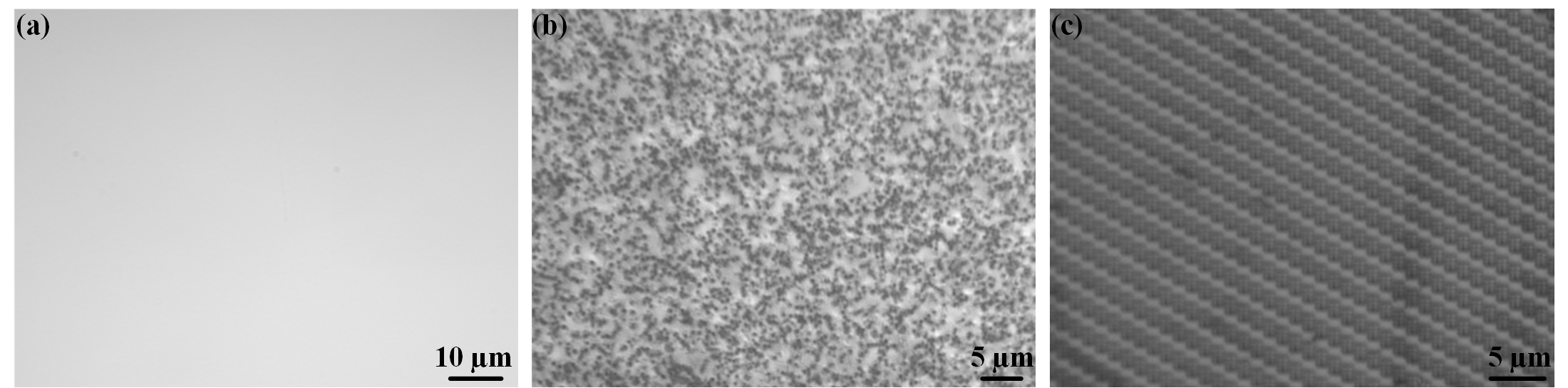


**Fig S5:** (a) Bright-field image of the BD sample acquired using a standard upright optical microscope equipped with a 100× objective lens. (b) Image of a chip circuit structure captured under the same 100× objective. (c) Image of the AAO sample obtained with the 100× objective.

To access the stability and reproducibility of the ultra-microscopic objective (UO) in super-resolution imaging applications, multiple imaging tests were performed on chip circuit structures and silver nanowire samples. The UO was constructed by combining a 63× water-immersion objective lens with a TBY microsphere-based plano-convex lens. UO units numbered 1, 2, and 3 were employed to evaluate imaging stability. Fig. S6(a-i) presents a super-resolution image of the chip circuit structure acquired using UO 1, with the corresponding intensity profile along the yellow horizontal arrow shown in Fig. S6(a-ii). The same UO was subsequently used to image a silver nanowire sample, as shown in Fig. S6(a-iii), where the calculated magnification factor is indicated in the bottom left corner. Figs. S6(b) and S6(c) display super-resolution images and their corresponding intensity profiles obtained using UO units 2 and 3, respectively. The consistent imaging results obtained from different samples using the same UO demonstrate its outstanding stability and reproducibility. Significantly, as the circuit structure is confined to a very small area on the chip and the silver nanowires are randomly distributed, UO-assisted imaging enables precise localization of target regions and supports repeatable detection of the samples. Further imaging of AAO samples (Fig. S6(d)) confirms the system’s robust capability to achieve super-resolution imaging across various types of specimens. Additionally, a UO assembled using a 50× air objective lens from the Olympus system was successfully integrated into different microscope platforms, highlighting the excellent compatibility and adaptability of the UO design (see Fig. S6(e)).


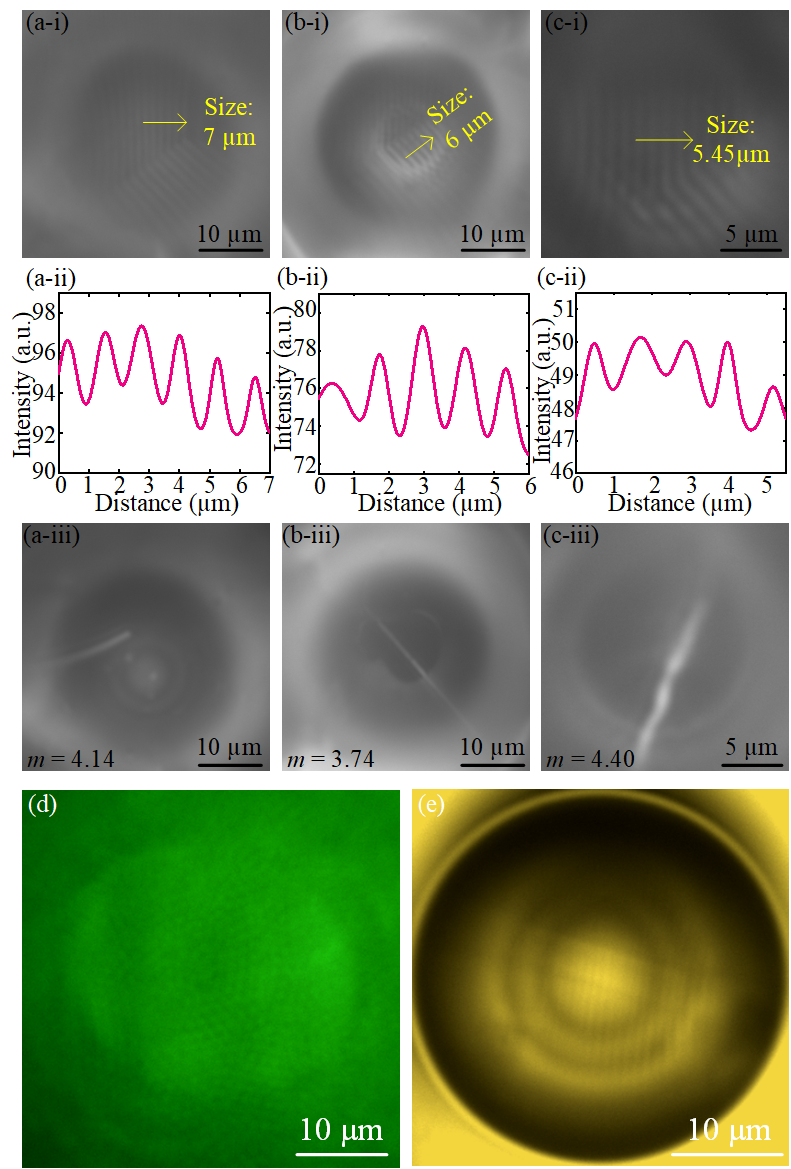


**Fig. S6:** (a) Super-resolution imaging of chip circuit structures and silver nanowire samples using UO 1, which integrates a 63× water-immersion objective lens with a TBY microsphere plano-convex lens: (i) super-resolution image of the chip circuit structure, (ii) intensity profile corresponding to the yellow arrow in (i), (iii) image of the silver nanowire sample with the magnification factor indicated in the bottom right corner. (b–c) Super-resolution images and their corresponding intensity profiles of the same samples obtained using UO units 2 and 3, respectively. (d) Super-resolution image of an AAO sample captured using a UO system equipped with a 63× water-immersion objective. (e) Super-resolution image of a BD sample acquired using a UO system composed of an Olympus 50× air objective lens integrated into a Zeiss microscope platform.

**Table S1.** Quantitative comparison of the proposed UO with representative microsphere-assisted imaging designs.

|  | **Effective FoV fraction** | **Magnification** | **Reference** |
| --- | --- | --- | --- |
| SMAL | ~40% | ~3× to ~4.36× | [1] |
| Overslip Superlens | ~25% | ~3× to ~3.5× | [2] |
| PCM | ~21-28% | ~3.03× to ~4.95× | [3] |
| UO | ~25–53% | ~3× to ~4.5× | this work |

**Reference**

[1] S. L. Stanescu, S. Vilain, D. Lonsdale, and L. Li, “Immersion-free non-contact imaging of sub-wavelength nanostructures down to 55 nm with super-resolution microsphere amplifying lens (SMAL),” in *OSA Imaging and Applied Optics Congress 2021 (3D, COSI, DH, ISA, pcAOP), paper IF2H.4*, DC: Optica Publishing Group, 2021.

[2] K. W. Allen *et al.*, “Super-resolution microscopy by movable thin-films with embedded microspheres: Resolution analysis,” *Ann. Phys.*, vol. 527, no. 7–8, pp. 513–522, 2015.

[3] B. Yan, Y. Song, X. Yang, D. Xiong, and Z. Wang, “Unibody microscope objective tipped with a microsphere: Design, fabrication, and application in subwavelength imaging,” *Appl. Opt.,* vol. 59, no. 8, pp. 2641–2648, 2020.

1. ***Corresponding author:** **Guoqiang Gu and Hui Yang**, Shenzhen Institutes of Advanced Technology, Chinese Academy of Sciences, Shenzhen, China; Stay Key Laboratory of Biomedical Imaging Science and System, Key Laboratory of Biomedical Imaging Science and System, Chinese Academy of Sciences, Shenzhen , China; and University of Chinese Academy of Sciences, Beijing, China; E-mail: [gq.gu@siat.ac.cn](mailto:gq.gu@siat.ac.cn) (Guoqiang Gu); <https://orcid.org/0000-0001-9853-5799>; [hui.yang@siat.ac.cn](mailto:hui.yang@siat.ac.cn) (Hui Yang); <https://orcid.org/0000-0002-6800-6308>; **Shengchuang Bai**, Laboratory of Infrared Material and Devices, Advanced Technology Research Institute, Ningbo University, Ningbo, China; [baishengchuang@nbu.edu.cn](mailto:baishengchuang@nbu.edu.cn); https://orcid.org/0000-0003-1478-5702.

   **Haonan Zhuo, Zhenmin Wang:** Shenzhen Institutes of Advanced Technology, Chinese Academy of Sciences, Shenzhen, China; Laboratory of Infrared Material and Devices, Advanced Technology Research Institute, Ningbo University, Ningbo, China

   **Zhouyi Yu, Duanduan Wu, Xunsi Wang:** Laboratory of Infrared Material and Devices, Advanced Technology Research Institute, Ningbo University, Ningbo, China

   **Zejie Zheng:** Shenzhen Institutes of Advanced Technology, Chinese Academy of Sciences, Shenzhen, China; College of Physics and Optoelectronic Engineering, Shenzhen University, Shenzhen, China

   **Yu Zhuang, Yina Jiang, Tiaoyao Zhang, Hao Li:** Shenzhen Institutes of Advanced Technology, Chinese Academy of Sciences, Shenzhen, China

   **Hao Li, Lixiang An:** National Innovation Center for Advanced Medical Devices, Shenzhen, China [↑](#footnote-ref-1)
